# Trace Level Analysis of Per- and Polyfluorinated Substances in Fish from Various Regions in Switzerland

**DOI:** 10.3390/toxics11110909

**Published:** 2023-11-07

**Authors:** Alexandra Jaus, Peter Rhyn, Judit Valentini

**Affiliations:** 1 Swiss Federal Institute of Metrology, Lindenweg 50, CH-3003 Bern-Wabern, Switzerland; 2 Federal Food Safety and Veterinary Office, Schwarzenburgstrasse 155, CH-3003 Bern, Switzerland; judit.valentini@blv.admin.ch

**Keywords:** PFAS, PFOS, fish muscle meat, SPE HPLC-MS/MS

## Abstract

Per- and polyfluorinated substances (PFASs) are persistent man-made chemicals which can end up in the food chain. In this study, the concentrations of 15 PFASs in various wild fish species from different regions in Switzerland were determined excluding hot spots of contamination. After clean-up with SPE, the samples were analyzed by HPLC-MS/MS. PFASs were detected in all but 1 of the 83 fish samples (0.07 to 40.7 µg/kg fish muscle meat). The most abundant compound in fish from subalpine lakes was perfluorooctane sulfonic acid (PFOS), comprising more than 80% of the total contamination while perfluorononanoic (PFNA), -decanoic (PFDA) and -undecanoic (PFUnDA) acid dominated in high alpine fish. PFAS levels were more elevated in subalpine lakes (median PFASs 11.1–19.0 µg/kg) than in the high alpine Lake Sils (median PFASs 0.66–2.67 µg/kg) or streams and canals in Valais (median PFASs 0.56 µg/kg). Our results indicate that wild fish may be one of the PFAS sources in human diet.

## 1. Introduction

PFASs comprise a wide class of chemicals. Many of them have unique properties, such as water and oil repellence and high biological, chemical and thermal stability. Therefore, PFASs are used in almost all industry branches and in many consumer products [1]; for example, in the textile, paper or leather industries. In addition, PFASs are used as wetting agents in electroplating and in refrigerants, blowing agents and fire-fighting foams.

PFASs are of concern because of their extremely high persistence, combined with bioaccumulation or mobility as well as toxicity [2]. Consequently, some of the most important PFASs were listed in the Stockholm Convention as new persistent organic pollutants (POPs) to be phased out like perfluorooctanoic acid (PFOA) and perfluorohexane sulfonic acid (PFHxS) or restricted in use (PFOS) [3]. Subsequently, the industry has shifted towards polyfluorinated PFASs with unknown toxicities and environmental fates, such as 3*H*-perfluoro-3-[(3-methoxy-propoxy)propanoic acid] (ADONA) or hexafluoropropylene oxide-dimer acid (HFPO-DA) [4].

Diet is the primary non-occupational intake pathway for human exposure to PFASs, and, besides other food of animal origin, fish may contain important concentrations and can thus be a dietary source of PFASs [5,6]. Therefore, the EU recently published maximum levels for PFOA, PFNA, PFHxS and PFOS in the muscle meat of fish ranging from 0.2 to 35 µg/kg muscle meat for individual substances and from 2 to 45 µg/kg for the sum of these four PFASs depending on the fish species [7]. The regulation refers to the tolerable weekly intake of 4.4 ng per kg of body weight for the sum of the four PFASs mentioned above which was established by the European Food Safety Authority in 2020 [8]. The values are set at a strict level which is reasonably achievable by following good agricultural, fishery and manufacturing practices and taking into account the risk related to the consumption of the food [7]. Furthermore, in 2013, the European commission set an Environmental Quality Standard (EQS) within the Water Framework Directive for PFOS in biota EQS_biota_ of 9.1 µg/kg wet weight to protect human health via consumption of fishery products [9]. Currently, this EQS_biota_ is under revision and a more stringent value for the sum of 24 PFAS has been proposed [10].

The main aim of the current study was to provide a data set of current PFAS contamination in fish from several areas in Switzerland ranging from high alpine areas down to subalpine lakes. A second aim was to set our results in relation with EU maximum levels to assess the importance of further studies on PFAS contamination.

The 15 target compounds were perfluorinated carboxylic acids, perfluorinated sulfonic acids and polyfluorinated alternatives. An overview of the investigated PFASs and their acronyms is given in Table S1.

## 2. Materials and Methods

### 2.1. Fish Samples

A total of 30 fish samples were obtained from Lake Sils in Grisons (*n* = 10 samples each from trout, char, grayling), 13 samples from 13 various local streams and canals in Valais (*n* = 12 trout, *n* = 1 chub) and 40 samples altogether were from Lake Geneva (*n* = 4 whitefish, *n* = 7 perch, *n* = 2 pike, *n* = 3 roach, *n* = 2 char, *n* = 1 tench), Lake Neuchatel (*n* = 10 whitefish, *n* = 5 perch, *n* = 1 pike, *n* = 1 trout, *n* = 1 pikeperch) and Lake Joux (*n* = 1 whitefish, *n* = 1 perch, *n* = 1 roach) in the western part of Switzerland. For all fish samples, the muscle meat was separated and roughly cut into pieces.

### 2.2. Chemicals and Reagents

All PFAS standards and internal standards are listed in Table S1. The solutions were obtained from Wellington Laboratories and stored at −20 °C. Working solutions of all analytes or internal standards (100 ng/mL) were prepared in methanol and stored at 5 °C for a maximum of 1 month.

Polypropylene pipet tips for Gilson Microman were used for all volumetric transfers and polypropylene vials with polypropylene caps (Fisher Scientific, Reinach, Switzerland) for instrumental analysis. Solvents and solutions intended for sample preparation were first tested for the absence of the analytes.

### 2.3. Sample Preparation

All lab equipment was previously rinsed with methanol.

The whole fish muscle sample was homogenized with five times the amount of water (Polytron, Kinematica, Malters, Switzerland) and aliquots of 12 g (corresponding to 2 g fish muscle meat) were transferred into a 30 mL polypropylene tube. Samples were prepared according to Sadia et al. [11] with the following minor modifications: 12 g of fish slurry were spiked with 1 ng of each internal standard and additionally 10 ng of ^13^C_4_-PFOS, 2.0 mL of aqueous 1.0 M sodium hydroxide solution were added to the slurry and after ultrasonic treatment neutralization was performed with 2.0 mL of 1.0 M hydrochloric acid.

### 2.4. HPLC-MS/MS Conditions

The analysis was performed on a Spark Symbiosis HPLC System (Axel Semrau, Sprockhoevel, Germany) coupled to a Sciex API 5000 MS/MS.

For chromatographic separation, a Zorbax Eclipse XDB-C8 column (3.5 μm, 3.0 × 100 mm, Agilent, Waldbronn, Germany) was used. The column temperature was 30 °C and the flow rate was 0.7 mL/min. Eluent A was 10 mM ammonium acetate buffer pH 4.3/methanol/acetonitrile (90/5/5, *v*/*v*/*v*), eluent B methanol/acetonitrile (50/50, *v*/*v*) and the following gradient program was applied: 0 min, 10% B; 1:00 min, 10% B; 1:30 min, 45% B; 7:30 min, 90% B; 8:00 min, 90% B. To minimize possible contamination with PFASs from the eluent or HPLC system, a trap column (Agilent Zorbax Eclipse XDB-C8 5 μm, 2.1 × 50 mm) was installed between the pump and the injector switching valve.

Autosampler needle rinsing was conducted as follows: 700 µL of water, 700 µL of methanol/acetonitrile 1:1 (*v*/*v*), 300 µL of water, 700 µL of water/methanol 1:1 (*v*/*v*) and finally 700 µL of water.

MS/MS quantification was performed with electrospray ionization in negative mode applying scheduled multiple reaction monitoring (sMRM, MRM detection window 25 s, target scan time 0.7 s). Two MRM transitions with optimum signal-to-noise ratios were monitored per analyte; the one with the highest signal intensity was selected as the quantifier (Table S1).

Chromatographic retention of the first eluting compound PFBA was adjusted to >2.5 min in order to discard the remaining sample matrix by a diverter valve. A chromatogram of a spiked perch sample is given in Figure S1.

### 2.5. Calibration and Quantitation

A 6-point calibration curve (0.02 to 2.0 µg/kg for analytes, 0.5 µg/kg for all internal standards) was freshly prepared in methanol every day.

For PFOS, two internal standards were used: ^13^C_8_-PFOS was added to the samples in a concentration of 0.5 µg/kg while the ^13^C_4_-PFOS concentration was 5 µg/kg. Samples with a PFOS concentration above the calibration range could be quantified with ^13^C_4_-PFOS as the internal standard.

For branched PFOS, the isomers were quantified separately with the calibration curve for linear PFOS and the concentrations for the *m*/*z* 499 > 80 and 499 > 99 product ions were averaged, as described in Riddell et al. [12] and Gyllenhammar et al. [13].

### 2.6. Method Validation and Quality Control

The established method was validated by determining linearities, limits of detection (LODs), limits of quantitation (LOQs), recoveries and precisions. Detailed validation data can be found in Table S2. Linear calibration curves (1/x weighted) were created for each analyte by plotting the area ratios versus the concentration ratios. Inter-day precision was determined with an in-house quality control sample (perch fortified with 0.5 µg/kg of all analytes) worked up with every sequence. Reproducibility was determined by analyzing certified reference material IRMM-427 (European Commission Joint Research Centre) or FAPAS QC material T0687QC (Fera Science) with every analytical series. Generally, recoveries of the analytes were 90–110% with relative standard deviations <10%. The LODs were 0.01–0.02 µg/kg and LOQs were 0.02–0.05 µg/kg. Thus, the LOQs fulfilled the sensitivity requirements of LOQ ≤ 0.1 µg/kg in the current EU recommendation regarding the monitoring of perfluoroalkyl substances in food [14].

Final sample solutions were stable for at least 48 h when storing the vials at 10 °C in the dark.

## 3. Results

**Figure 1:**
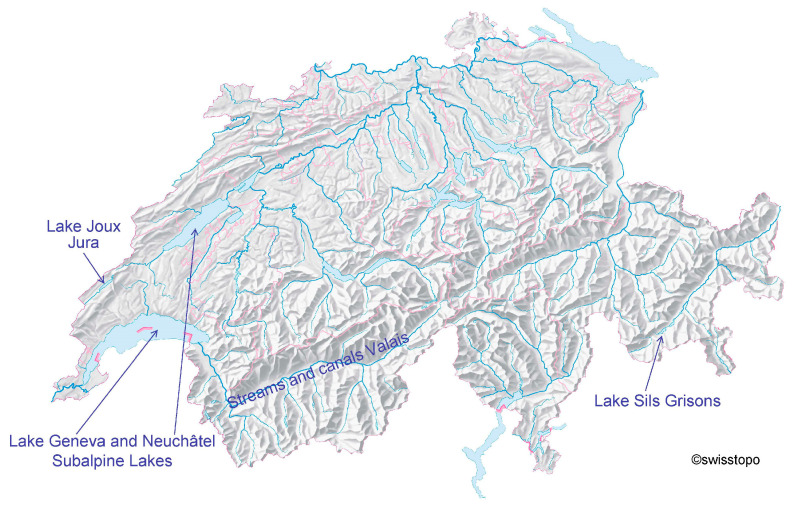
Map of the fish sampling regions in Switzerland.

Contamination of wild fish species with PFASs was determined for different regions of Switzerland: the sampling sites ranged from the high alpine Lake Sils in the canton of Grisons over alpine and subalpine streams and canals in the canton of Valais and Lake Joux, in the medium mountain range Jura, down to the large subalpine lakes Geneva and Neuchâtel in western Switzerland which are densely populated areas and surrounded by agriculture and industry. The sampling regions are illustrated in Figure 1. No samples originating from areas highly contaminated with PFAS are reported here. Due to the different fish habitats, it was not possible to systematically obtain samples of the same fish species from various waters.

Table 1 provides an overview of the fish species and the PFAS concentrations observed in the different sampling regions. PFNA, PFDA, PFUnDA and PFOS were detected in the vast majority of the investigated samples, PFOA in 15% of the samples, while PFBA, PFPeA, PFHxA, PFHpA, PFBS, HFPO-DA, ADONA and 9Cl-PF3ONS were never detected. PFHxS and 8:2 FTS were found in less than 10% of the samples and at maximum concentrations of 0.1 µg/kg. Therefore, these compounds are not discussed any further. For the individual samples, the PFAS sum ranged from <0.02 to 40.7 µg/kg (*n* = 83). Branched PFOS isomers were observed at levels up to 5.1% of total PFOS.

In fish from the high alpine Lake Sils, PFOA, PFNA, PFDA and PFUnDA were observed at comparable or slightly higher levels than in the subalpine lakes, while the overall contamination with PFASs was clearly lower (Lake Sils 0.19–7.04 µg/kg, subalpine lakes 1.15–40.7 µg/kg). Fish from alpine and subalpine streams and canals in Valais excluding PFAS contamination hotspots revealed comparatively low PFAS levels (<0.02–6.95 µg/kg).

Although there was considerable variation in concentrations from sample to sample and among species within the same sampling region (see Table 1), generally more important differences resulted between the median fish concentrations in samples from the subalpine Lakes Geneva and Neuchâtel compared to the other sampling regions.

**Figure 2:**
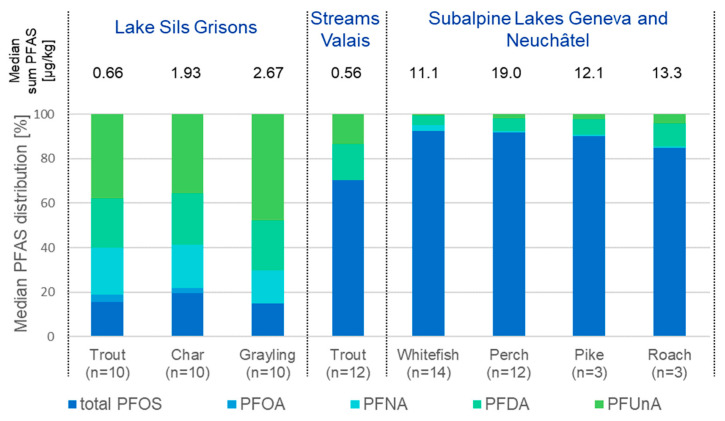
Median sum PFASs and percentage distribution for various fish species and sampling regions. Only species with at least three samples per region were included.

The distribution patterns of the detected PFASs for fish species in the different sample regions (median values for each component) as well as the median PFAS concentrations are illustrated in Figure 2. In the high alpine Lake Sils in Grisons, the long-chain carboxylic acids (PFOA, PFNA, PFDA and PFUnDA) accounted for at least 80% of the PFASs, while in the streams and canals of Valais and in the subalpine lakes the PFAS, distribution was the inverse with PFOS largely dominating (70% in Valais, >85% in subalpine lakes).

## 4. Discussion

Generally, PFAS levels were higher in the subalpine lakes compared to alpine areas, which had been observed previously [15]. PFBA to PFHpA and PFBS were not detected in any sample, which could be explained by a decrease in bioaccumulation with decreasing perfluorinated chain length [15,16]. Although the observed proportion of up to 5% of branched PFOS isomers of the total PFOS concentration was slightly lower than the value of 8% observed in Sweden [17], it confirms that the branched/linear PFOS isomer proportion present in fish is clearly lower than observed in the technical mixture (PFOS produced by electrochemical fluorination processes and yielding about 77% linear PFOS and 23% branched isomers) or in drinking water (44–57% branched) [18].

Published data on PFAS contamination in fish from Switzerland are scarce and comparison was only possible when also including other fish species and different lakes: Valsecchi et al. [15] observed a median value of 9.1 µg/kg PFOS and 11.7 µg/kg sum PFASs in 14 fillet samples (roach and burbot) from Lake Geneva; PFNA, PFDA, PFUnDA and PFHxS were detected in amounts of 0.9–0.81 µg/kg. Furthermore, for Lago Maggiore and Lago di Lugano, two large Italian–Swiss subalpine lakes, they reported overall PFOS median values of 8.6 and 15.7 µg/kg as well as overall PFASs median results of 10.2 and 18.6 µg/kg. In Lake Geneva, a median PFOS value of 34.9 µg/kg was observed for 74 whole fish samples (roach, burbot and perch) [19], corresponding to 19.3 µg/kg fillet (formula from [19]). In fish fillets from the German–Austrian–Swiss Lake Constance, Riemenschneider et al. [20] observed a median PFOS value of 11.63 µg/kg for whitefish (*n* = 80) and 9.87 µg/kg for perch (*n* = 40). PFNA, PFDA and PFUnDA were detected at median levels of 0.06 to 0.84 µg/kg. Recently, Wüthrich et al. [21] reported a median PFOS value in trout of 13 µg/kg (*n* = 50) with a maximum value of 49 µg/kg in various streams in the Swiss canton St Gallen.

Consequently, our results for fish in subalpine lakes (median PFOS 8.96–17.3 µg/kg, median PFASs 11.1–19.0 µg/kg) fit well within the framework of previously published PFAS values, despite partly differing fish species and sampling sites.

In the high alpine Lake Sils, PFOS was detected at low levels (median PFOS 0.10–0.52 µg/kg), but the predominant PFASs were PFNA, PFDA and PFUnDA.

Steingruber [22] analyzed trout and char from two interconnected high alpine lakes in an uninhabited area in the Swiss canton of Ticino (pool samples per species and lake). They reported PFOS values ranging from 0.20 to 0.86 µg/kg and generally dominating contributions of PFDA to perfluorotetradecanoic acid. This confirms the levels and distribution patterns observed in our study for fish from Lake Sils, both of which may be explained by the absence of direct sources and atmospheric transportation of their precursors [15,22].

Cross-country skiing, which is very popular in the Lake Sils region, may also be a potential local source of PFAS contamination [23]. However, our PFAS results for Lake Sils samples were very close to those published by Steingruber for an uninhabited alpine area [22], which indicates that ski wax was probably not a major source of PFAS fish contamination in Lake Sils.

Since none of the investigated species was completely herbivorous, no comparison of herbivorous and predatory fish was possible.

PFAS contamination levels of all samples were set in relation with the specified compound and species EU maximum levels [7], which are also intended to be implemented in Switzerland: all samples from Lake Sils and the investigated streams and canals in Valais fell largely below these levels, while PFOS contamination of four samples (two pikes, one trout and one perch) from subalpine lakes exceeded the limits. Compared to the EQS_biota_ of 9.1 µg PFOS/kg wet weight [9], the median results of the samples from subalpine lakes were close to or higher than this value, while the results of the other sampling regions were clearly below.

Consequently—although for the general population, wild fish contributes only a very small extent to the overall exposure with PFASs and therefore the occasional consumption of fish from subalpine lakes is not considered to be critical—further and more intensive monitoring of PFAS contamination in fish and other food is of major importance. Potential difficulties for further monitoring studies may, however, be the contradictoriness between the need for less complicated sample work-up and low detection limits required as well as—more specific for fish studies—the complexity of covering a vast area, numerous species and a large age spectrum.

## Figures and Tables

**Table 1:** PFAS levels for fish muscle meat of different species and sampling regions in Switzerland.

Sampling Region/Fish Species		PFOA [µg/kg]	PFNA [µg/kg]	PFDA [µg/kg]	PFUnDA [µg/kg]	PFOS [µg/kg]	Sum PFASs [µg/kg]
Lake Sils, Grisons							
Trout, *Salmo trutta* (*n* = 10)	% >LOQ	60	100	100	100	100	100
	Median (range)	0.06 (<LOD-0.36)	0.42 (0.08–1.78)	0.43 (0.17–1.63)	0.75 (0.36–2.50)	0.31 (0.14–0.77)	1.93 (0.86–7.04)
Char, *Salvelinus umbla* (*n* = 10)	% >LOQ	50	90	90	100	100	100
	Median (range)	0.06 (<LOD-0.26)	0.52 (<LOD-1.16)	0.61 (<LOQ-1.27)	0.95 (0.07–1.73)	0.52 (0.09–0.82)	2.67 (0.19–4.82)
Grayling, *Thymallus thymallus* (*n* = 10)	% >LOQ	10	80	90	100	90	100
	Median (range)	<LOD (<LOD-0.10)	0.10 (<LOD-0.36)	0.15 (<LOQ-0.41)	0.32 (0.15–0.82)	0.10 (<LOQ-0.26)	0.66 (0.24–1.95)
Lake Joux, Jura							
Whitefish, *Coregonus* sp. (*n* = 1)		<LOD	<LOD	0.08	0.08	0.54	0.73
Perch, *Perca fluviatilis* (*n* = 1)		<LOD	0.06	0.51	0.46	3.62	4.65
Roach, *Rutilus rutilus* (*n* = 1)		<LOD	<LOD	0.23	0.29	1.44	1.96
Streams and canals, Valais							
Trout, *Salmo trutta* (*n* = 12)	% >LOQ	8	17	58	58	92	92
	Median (range)	<LOD (<LOD-0.07)	<LOQ (<LOD-0.10)	0.07 (<LOD-1.06)	0.06 (<LOD-0.32)	0.31 (<LOD-5.32)	0.56 (<LOD-6.95)
Chub, *Squalius cephalus* (*n* = 1)		<LOD	<LOD	0.18	0.40	2.38	3.01
Subalpine Lakes Geneva and Neuchâtel							
Whitefish, *Coregonus* sp. (*n* = 14)	% >LOQ	14	100	100	100	100	100
	Median (range)	<LOQ (<LOD-0.06)	0.34 (0.17–0.61)	0.55 (0.33–1.14)	0.09 (0.06–0.23)	12.1 (5.01–26.2)	13.3 (5.58–28.2)
Perch, *Perca fluviatilis* (*n* = 12)	% >LOQ	0	100	100	100	100	100
	Median (range)	<LOD (all <LOD)	0.13 (0.07–0.23)	1.10 (0.91–2.24)	0.36 (0.26–0.69)	17.3 (9.31–37.7)	19.0 (10.8–40.7)
Pike, *Esox lucius* (*n* = 3)	% >LOQ	0	100	100	100	100	100
	Median (range)	<LOD (all <LOD)	0.07 (0.05–0.09)	0.76 (0.52–1.29)	0.27 (0.16–0.43)	9.98 (6.86–22.7)	11.1 (7.61–24.6)
Roach, *Rutilus rutilus* (*n* = 3)	% >LOQ	0	66.6	100	100	100	100
	Median (range)	<LOD (all <LOD)	0.07 (<LOD-0.08)	1.08 (0.27–2.00)	0.44 (0.12–1.04)	8.96 (3.75–12.9)	12.1 (4.14–14.6)
Char, *Salvelinus alpinus* (*n* = 2)		both <LOD	0.08/0.11	0.25/0.87	0.09/0.21	7.39/22.8	7.81/24.0
Trout, *Salmo trutta* (*n* = 1)		<LOD	0.16	1.14	0.31	17.2	18.9
Pikeperch, *Sander lucioperca* (*n* = 1)		<LOD	0.21	0.20	0.28	0.46	1.15
Tench, *Tinca tinca* (*n* = 1)		<LOD	0.34	1.68	0.66	5.58	8.37

## Data Availability

The data presented in this study are available on request from the corresponding author.

## Supplementary Materials

The following supporting information can be downloaded at: https://www.mdpi.com/article/10.3390/toxics11110909/s1, Table S1: Detailed names, acronyms, CAS-No of linear isomer, MS transitions (quantifier in bold) and internal standards for all analytes; Table S2: Detailed validation results for all analytes; Figure S1: HPLC-MS/MS chromatogram of a perch sample spiked with 0.5 µg/kg of all analytes. For better overview, the analyte traces are given in two graphs.
