# Supplementary material for: Trace Level Analysis of Per- and Polyfluorinated Substances in Fish from Various Regions in Switzerland
Source: Toxics. 2023 Nov 7;11(11):909. doi: 10.3390/toxics11110909 (PMC10675579; doi:10.3390/toxics11110909)
Supplement: Supplementary file 1 [file toxics-11-00909-s001.zip › toxics-2619774-supplementary.pdf]

toxics MDPI

Supplementary Materials: Trace Level Analysis of Per- and Polyfluorinated Substances in Fish from Various Regions in Switzerland

Alexandra Jaus, Peter Rhyn and Judit Valentini

Table S1. Detailed names, acronyms, CAS-No of linear isomer, MS transitions (quantifier in bold) and internal standards for all analytes.

Analyte	Acronym	CAS no	MS transitions	Internal Standard
Perfluorobutanoic acid	PFBA	375-22-4	213 > 169	$^{13}C_4$-PFBA
Perfluoropentanoic acid	PFPeA	2706-90-3	263 > 219	$^{13}C_5$-PFPeA
Perfluorohexanoic acid	PFHxA	307-24-4	313 > 269, 313 > 119	$^{13}C_5$-PFHxA
Perfluoroheptanoic acid	PFHpA	375-85-9	363 > 319, 363 > 169	$^{13}C_4$-PFHpA
Perfluorooctanoic acid	PFOA	335-67-1	413 > 369, 413 > 169	$^{13}C_8$-PFOA
Perfluorononanoic acid	PFNA	375-95-1	463 > 419, 463 > 219	$^{13}C_9$-PFNA
Perfluorodecanoic acid	PFDA	335-76-2	513 > 469, 513 > 219	$^{13}C_6$-PFDA
Perfluoroundecanoic acid	PFUnDA	2058-94-8	563 > 519, 563 > 269	$^{13}C_7$-PFUnDA
Perfluorobutane sulfonic acid	PFBS	375-73-5	299 > 99, 299 > 80	$^{13}C_3$-PFBS
Perfluorohexane sulfonic acid	PFHxS	355-46-4	399 > 99, 399 > 80	$^{13}C_3$-PFHxS
Perfluorooctane sulfonic acid	PFOS	1763-23-1	499 > 80, 499 > 99	$^{13}C_8$-PFOS/$^{13}C_4$-PFOS
Hexafluoropropylene oxide-dimer acid	HFPO-DA	62037-80-3	329 > 169, 285 > 169	$^{13}C_3$-HPFO-DA
3*H*-Perfluoro-3-[(3-methoxy-propoxy)propanoic acid]	ADONA	958445-44-8	377 > 251, 377 > 85	$^{13}C_3$-PFHxS
9-Chlorohexadecafluoro-3-oxanonane-1-sulfonic acid [1]	9Cl-PF3ONS/ 6:2 Cl-PFESA	756426-58-1	531 > 351, 531 > 83	$^{13}C_7$-PFUnDA
8:2 Fluorotelomer sulfonic acid	8:2 FTS	39108-34-4	527 > 507, 527 > 81	$^{13}C_2$-8:2 FTS

[1] 9Cl-PF3ONS is the main component of F-53B®

Table S2. Detailed validation results for all analytes.

Acronym	Repeatability 0.1 µg/kg [%], n=5	Repeatability 0.5 µg/kg [%], n=5	Inter-day precision 0.5 µg/kg [%] n=10	Reproducibility JRC-IRMM-427 [%], n=6 [1)]	Reproducibility FAPAS T0687QC [%], n=6 [1)]
PFBA	3.5	8.3	6.5	n/a	n/a
PFPeA	1.2	2.0	2.7	n/a	n/a
PFHxA	2.1	2.0	5.3	n/a	n/a
PFHpA	2.1	1.9	3.5	n/a	n/a
PFOA	2.3	1.7	3.0	n/a	3.1
PFNA	2.2	0.4	3.9	4.5	n/a
PFDA	0.9	2.5	5.2	4.4	n/a
PFUnDA	2.3	0.7	4.0	4.6	n/a
PFBS	1.8	1.8	3.0	n/a	n/a
PFHxS	2.0	1.5	4.6	6.0	n/a
PFOS	1.9	1.8	3.4	5.4	7.2
HFPO-DA	2.6	8.9	6.7	n/a	n/a
ADONA	6.8	5.4	2.8	n/a	n/a
9Cl-PF3ONS	3.5	1.7	7.7	n/a	n/a
8:2 FTS	3.0	4.4	6.1	n/a	n/a

Acronym	LOD/LOQ [µg/kg]	Linearity (1/x weighted): Regr. Coefficient R^2	Intra-day Recovery 0.1 µg/kg [%]	Intra-day Recovery 0.5 µg/kg [%]	Inter-day Recovery 0.5 µg/kg [%]
PFBA	0.02/0.05	0.993	123	114	104
PFPeA	0.02/0.05	0.992	96	100	100
PFHxA	0.02/0.05	0.990	100	105	101
PFHpA	0.02/0.05	0.996	98	100	100
PFOA	0.02/0.05	0.995	95	101	101
PFNA	0.02/0.05	0.996	98	100	100
PFDA	0.02/0.05	0.995	96	98	99
PFUnDA	0.02/0.05	0.996	116	105	105
PFBS	0.02/0.05	0.996	102	97	101
PFHxS	0.02/0.05	0.994	114	98	100
PFOS	0.02/0.05	0.991	99	103	96
HFPO-DA	0.01/0.02	0.998	94	100	97
ADONA	0.01/0.02	0.987	105	102	102
9Cl-PF3ONS	0.01/0.02	0.996	98	92	101
8:2 FTS	0.01/0.02	0.997	89	99	98

[(1)] These parameters could only be determined for those analytes with concentrations >LOQ in the certified reference material IRMM-427 or FAPAS QC material T0687QC
n/a: not applicable.

[(2)] The sensitivity was assessed by determining the signal-to-noise ratios (S/N) considering the S/N in the blank sample and the reagent blank (LOQ S/N 10:1, LOD S/N 3:1).

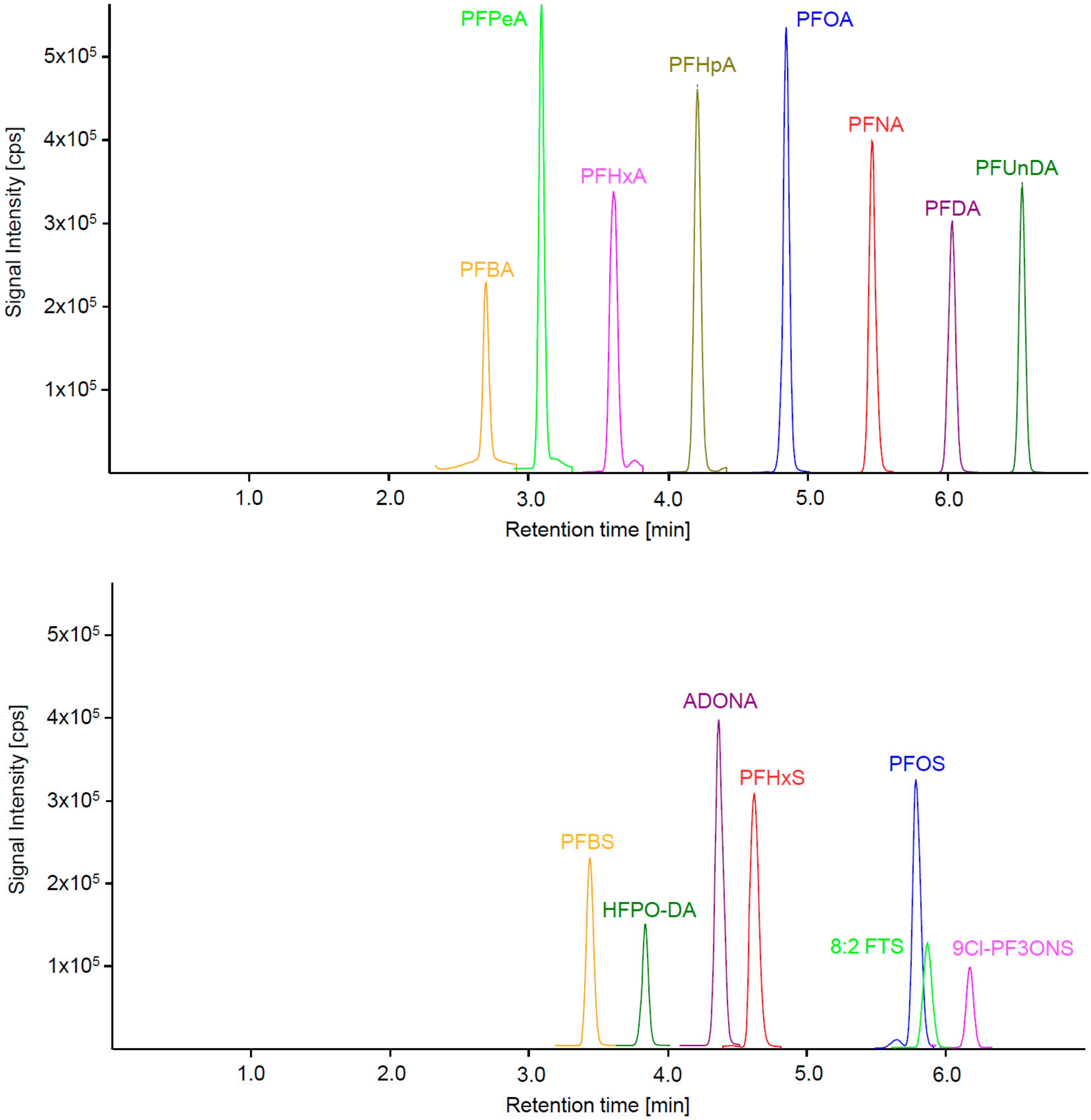

Figure S1. HPLC-MS/MS chromatogram of a perch sample spiked with 0.5 µg/kg of all analytes. For better overview, the analyte traces are given in 2 graphs.
